# Glycogen storage disorder types IX: the mutation spectrum and ethnic distribution

**DOI:** 10.1186/s13023-024-03488-0

**Published:** 2024-12-20

**Authors:** Bita Geramizadeh, Fatih Ezgu, Zahra Beyzaei

**Affiliations:** 1 Assistant Professor of Cellular and Molecular Medicine, Shiraz Transplant Research Center, Shiraz University of Medical Sciences, Khalili St., Research Tower, Seventh Floor, Shiraz, Iran; 2 Department of Pathology, Medical School of Shiraz University, Shiraz University of Medical Sciences, Shiraz, Iran; 3 Department of Pediatric Metabolism and Genetics, Faculty of Medicine, Gazi University, Ankara, Turkey

**Keywords:** Glycogen storage disease, Phosphorylase b Kinase, Genotype, Mutations

## Abstract

Glycogen storage disorders (GSD) GSD-IX are characterized by deficiencies in muscular and/or hepatic phosphorylase enzymes. GSD type IX za is an X-linked disorder, while IXb and IXc are autosomal recessive disorders resulting from pathogenic variants in the genes encoding the Phosphorylase b Kinase regulatory subunit alpha (*PHKA*), beta (*PHKB*), and gamma (*PHKG*), respectively. Despite progress in understanding these diseases, there are still unclear questions regarding their clinical manifestations, genetic variations, and the relationship between genotype and phenotype. Therefore, this review focuses on variants of GSD IX subtypes and all clinical findings to establish a genotype–phenotype relationship as well as highlighting the wide spectrum of disease-causing variants. Such information is beneficial for the establishment of a privileged mutation screening process in a specific region or ethnic group. Diagnosis is based on clinical manifestations and laboratory test results, but molecular analysis is often necessary to distinguish the various forms with similar presentations.

## Introduction

Glycogen storage disease type IX (GSD IX) results from a deficiency in phosphorylase b kinase, an enzyme with a vital regulatory role in the breakdown of glycogen. This deficiency in Phosphorylase kinase (PhK) can lead to the development of GSD IX, a condition characterized by impaired glycogen metabolism [1].

The enzyme PhK is composed of four copies, from four subunits including α, β, γ, and δ. There are two types of PhK deficiency: liver PhK deficiency, which typically manifests in early childhood with symptoms such as hepatomegaly, growth restriction, hypoglycemia and occasionally fasting ketosis; and muscle PhK deficiency, a less common form characterized by myalgia, muscle cramps, exercise intolerance, and progressive muscle weakness [2]. While it was previously thought that symptoms and biochemical abnormalities of liver PhK deficiency would improve with age, recent evidence suggests the need for long-term monitoring of affected patients due to the risk of complications such as liver fibrosis, cirrhosis, and more severe manifestations. It is now evident that the condition should be taken seriously to ensure appropriate care and management [3, 4].

The various subtypes of GSD IX are linked to pathogenic variants in *PHKA1, PHKA2, PHKB*, and *PHKG2* genes [5]. By understanding the relationship between these pathogenic variants and the subtypes of GSD IX, we can obtain valuable insight into the underlying mechanisms of the disease. The three subtypes of this condition with liver involvement, caused by pathogenic variants in three different genes named: *PHKA2, PHKB*, and *PHKG2*, which cannot be differentiated based on their clinical features, while the severity of symptoms can vary significantly. Typically, children with this condition present in their early years with symptoms like hepatomegaly and growth restriction, which are similar to other types of glycogen storage diseases such as GSD I, III, IV, and VI [6, 7]. Ketotic hypoglycemia, if present, is generally mild but can occasionally be severe and recurrent [8, 9]. Therefore, the differentiation of subtypes is challenging.

This comprehensive review aims to provide an overview of the diverse variants found in GSD IX subtypes among different ethnic groups. Additionally, we will explore the impact of these alterations on the clinical presentation. Our goal is to provide a comprehensive understanding of the subject matter and shed light on the correlation between genetic variations and clinical outcomes.

## Phosphorylase b kinase regulatory subunit alpha, skeletal muscle isoform (*PHKA1*) gene

### Gene

*PHKA1* is a gene located on the Xq13.1 region, responsible for encoding the muscle isoform of subunit α which consists of a 1,223-amino-acid protein. Mutations in this gene lead to a rare X-linked disorder known as muscle PhK deficiency. The *PHKA1* gene spans approximately 133 kb of genomic DNA and is comprised of 32 exons, which are transcribed into a 6-kb cDNA. Understanding the structure and characteristics of the *PHKA1* gene is crucial in unraveling the mechanisms underlying muscle PhK deficiency [10, 11].

### Variants

**Fig. 1:**
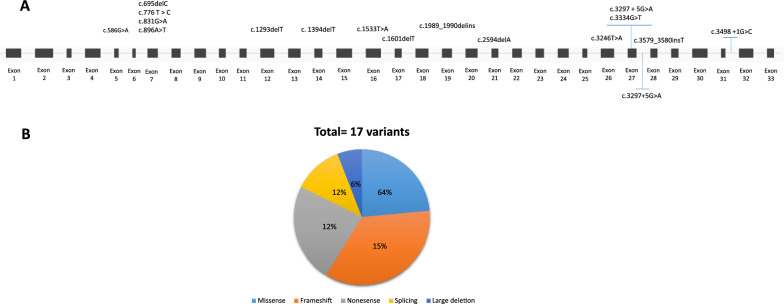
Schematic illustration (not to scale) of the *PHKA1* gene and location of sequence variants associated with Glycogen storage disease type IXa1 deficiency. **A**: Exon (boxes)–intron (lines) structure of the human *PHKA1* gene. Coloring boxes are consistent with panel **B**: Pie chart summarizing types of *PHKA1* variants reported to date

To date, 17 pathogenic variants in *PHKA1* from 20 families (22 patients) have been reported, each has been found in only one or two individuals. Pathogenic variants include frameshift (n = 6), missense (n = 4), nonsense (n = 4), splice site changes (n = 2), and large deletion (n = 1). Fifteen of them are in the exonic region and 2 out of 17 variants are intronic ones (Fig. 1A, B). Frameshift variants illustrate the predominant variant type (n = 11). A large deletion, resulting in the skipping of two complete exons (Exons 29 and 30), has been reported [11]. Moreover, two intronic splicing sites in the intron also has been observed. All variants are unique, except c. 1394delT and c.695delC, which are deletion variants found in 4 different patients from 4 families [12–14]. The reported variants have been observed in patients from diverse ethnic backgrounds, including European, North American, Asian, and Australian populations, indicating a wide geographical distribution [10, 15–17]. At present, there is limited information available regarding the epidemiology and molecular defects in *PHKA1* patients. To the best of our knowledge, no variant in the *PHKA1* gene has been reported from the Middle East, Central America, Africa, or the Oceania continent (Table 1).

**Table 1:** Summary of reported variants and clinical manifestations in *PHKA1* gene of patients with GSD IXa1

Variant	Variant type	Clinical presentation	No. patients	Type of variant	Exon/Intron	Country	Study
c.586G>A	Glu196Lys	Myalgia, fatigue	1	Missense	E5	Denmark	Andersen et al. [16]
c.695delC	Ala232fs	Myalgia, elevated CK, muscle pain	3	Frameshift	E7	Belgium, Denmark	Wuyts et al. [14]; Preisler et al. [13]
c.776 T>C	–	–	1	Missense	E7	Russia	Savostyanov et al. [59]
c.831G>A	–	Exercise intolerance, nighttime muscle cramps	1	Missense	E7	Denmark	Ørngreen et al. [76]
c.896A>T	Asp299Val	Exercise intolerance, Myopathy	2	Missense	E7	Germany	Burwinkel et al. [39, 46]
c.1293delT	Phe430fsTer	Mild exercise-induced forearm pain	1	Frameshift	E12	Denmark	Preisler et al. [13]
c. 1394delT	Thr464fsTer	Elevated CK, Mild myopathy	2	Frameshift	E14	USA, France	Echaniz-Laguna et al. [12]
c.1533 T>A	Tyr511Ter	Gradual muscle weakness and Myalgia of all four limbs, elevated CK	1	Nonsense	E16	China	Huang et al. [11]
c.1601delT	Leu534Argfs*5	Severe childhood speech disorder	1	Frameshift	E17	Australia	Hildebrand et al. (2020)
c.1989_1990delins AAGTTGCTCGTGATCTAAA	Tyr663Ter	Progressive muscle weakness, dysarthria, chewing and swallowing difficulties, chronic respiratory failure, abnormal muscle pain	1	Frameshift	E19	China	Wang et al. [15]
c.2594delA	Lys865Argfs*15	A progressive myopathy, exercise intolerance, muscle hypertrophy, and cognitive impairment	2	Frameshift	E21	France	Bisciglia et al. [10]
c.3246 T>A	Cys1082Ter	Muscle pain and elevated CK	1	Nonsense	E26	China	Li et al. [17]
c.3297+5G>A		muscle weakness of the lower limbs and gradual loss of the ability to climb stairs, high CK levels	1	Splice site	E27	China	Huang et al., [11]
c.3334G>T	Glu1112Ter	Gait disturbance, myopathy	1	Nonsense	E27	Germany	Wehner et al. [74]
c.3579_3580insT	Ser1194Ter	Myalgia	1	Nonsense	E28	Japan	Munekane et al. [75]
c.3670_3924del255	–	Muscle weakness, moderately high CK levels	1	Large deletion	Skipping E29-30	China	Huang et al. [11]
c.3498+1G>C	–	Exercise intolerance, with cramps and weakness of exercising muscles, myoglobinuria	1	Splice site	I31	USA	Bruno et al. (1998)

### Genotype–phenotype correlation

The reported variants in patients have been found to be associated with various clinical manifestations, including myalgia (31.8%), myopathy (22.7%), elevated creatine kinase (CK) levels (22.7%), exercise intolerance (18.2%), and muscle weakness (22.7%). Additionally, there have been rare reports of severe childhood speech disorders, chewing and swallowing difficulties, chronic respiratory failure, cognitive impairment, gait disturbance, and myoglobinuria. However, given the limited number of patients, it is currently not possible to establish a definitive genotype–phenotype correlation. Further investigation and research are needed to gather more conclusive evidence in this regard. To date, there have been no reported cases of liver transplants in these patients.

## Phosphorylase b kinase regulatory subunit alpha 2 (*PHKA2*) gene.

### Gene

The most common form of liver PhK deficiency, known as X-linked liver glycogenosis, is caused by mutations in the *PHKA2* gene. This gene, located on Xp22.13, consists of 33 exons and spans 91.3 kb of DNA. *PHKA2* encodes the liver alpha subunit of PhK. The mRNA, spanning 5,325 base pairs, is translated into a protein consisting of 1,235 amino acids. This protein is primarily expressed in the liver and brain, with minimal expression in muscle tissue [18–20]. It is worth noting that *PHKA2* shares a high degree of homology with *PHKA1* and *PHKB* [21].

### Variants

**Fig. 2:**
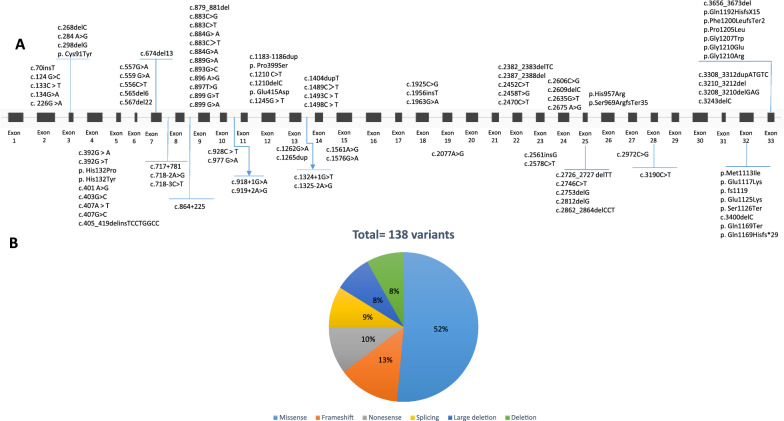
Schematic illustration (not to scale) of the *PHKA2* gene and location of sequence variants associated with Glycogen storage disease type IXa2 deficiency. **A**: Exon (boxes)–intron (lines) structure of the human *PHKA2* gene. Coloring boxes are consistent with panel **B**: Pie chart summarizing types of *PHKA2* variants reported to date

A total of 138 different pathogenic variants have been reported in the *PHKA2* gene, identified in 223 patients. Among these variants, the majority are missense variants (70 out of 138), followed by frameshift (n = 18), nonsense (n = 14), splice site (n = 12), large deletion (n = 11), deletion (n = 11), and duplication (n = 2) variants (Fig. 2A, B). These pathogenic variants are distributed throughout the gene, with 18 variants occurring in intronic regions and the remaining variants found in exonic regions, spanning most of the *PHKA2* exons. The two most frequently observed variants, Arg295His and Pro1205Leu, have been reported in a total of 11 patients from diverse ethnic populations [20, 22–32]. Additionally, a few other variants have been reported in four or three related patients, while each of several others have been documented in only one patient (Table 2).

**Table 2:** Summary of reported variants and clinical manifestations in *PHKA2* gene of patients with GSD IXa2

Variant	Variant type	Clinical presentation	No. patients	Type of variant	Exon/Intron	Country	Study
c.4C>G	Arg2Gly	Afebrile fasting episodes of vomiting, shakiness, uneasiness, drowsiness, relief after food, IKH	4	Missense	3`UTR	Denmark	Benner et a. [34]; Flejsborg et al. [73]
c.70insT	fs24	Elevated serum ALT	1	Frameshift	E2	Japan	Burwinkel et al. (1998)
c.124 G>C	Ala42Pro	Hepatomegaly	1	Missense	E2	France	Davit-Spraul et al. [29]
c.133C>T	Arg45Trp	Hepatomegaly, Growth retardation	4	Missense	E2	China, Russia, USA	Zhu et al. [18]; Zhang et al. [23], Savostyanov et al. [59]; Wang et al. (2012)
c.134G>A	Arg45Gln	Hepatomegaly	2	Missense	E2	India, China	Kumar et al. [55]; Zhang et al. [23]
c. 226G>A	Glu76Lys	Periodic hyperglycemia	2	Missense	E2	Russia	Kamenets et al. [54]; Bolotova et al. (2017)
c.268delC			1	Missense	E3	Russia	Kamenets et al. [54]
	Cys91Tyr	Hepatomegaly, elevated serum ALT	1	Missense	E3	Czech	Rudolfova et al. [66]
c.284 A>G	Gln95Arg	Hepatomegaly	1	Missense	E3	France	Davit-Spraul et al. [29]
c.298delG	fs100	Hepatomegaly, delayed growth in childhood, elevated transaminases	1	Frameshift	E3	UK	Hendrickx et al. [69]
c.338A>G	His113Arg	Hepatomegaly	1	Missense		China	Zhang et al. [23]
c.345_359dup15	Tyr116_ Thr120dup	Hepatosplenomegaly, Mild motor delay, muscle weakness and fatigue	1	Duplication		UK	Beauchamp et al. [44]
	Tyr116Asn		1	Missense		Japan	Hirono et al. [31]
c.363C>T	Thr121Met	Hepatomegaly, elevated transaminases	2	Missense		Korea	Sohn et al. (2017)
c.392G>A	Gly131Asp	Hepatomegaly, elevated transaminases	2	Missense	E4	China, France	Zhang et al. [23]; Davit-Spraul et al. [29]
c.392G>T	Gly131Trp	hepatomegaly, developmental delay, poor muscle control, elevated transaminases	1	Missense	E4	USA African-American	Khan et al. [36]
c.392 G>T	Gly131Val	Hepatomegaly	1	Missense	E4	France	Davit-Spraul et al. [29]
	His132Pro		1	Missense	E4	Germany	Burwinkel et al. [51]
	His132Tyr		1	Missense	E4	Germany	Burwinkel et al. [51]
c.401 A>G	Gln134Arg	Hepatomegaly	1	Missense	E4	France	Davit-Spraul et al. [29]
c.403G>C	Val135Leu	Mostly morning afebrile attacks of hypotonia, sweating, drowsiness, shakiness, vomiting, relief after food	1	Missense	E4	Denmark	Benner et a. [34]
c.407A>T	Asp136Val	Hepatomegaly, elevated transaminases, Frequent hunger Fatigue	1	Missense	E4	China	Zhang et al. [23]
c.407G>C	Asp136Profs		1	Frameshift	E4	South Korea	Sohn et al. (2019)
c.405_419delinsTCCTGGCC	Asp136ProfsTer11	Hepatomegaly and elevated serum ALT	1	Frameshift	E4	South Korea	Kim et al. (2020)
c.508 T>G	Phe170Val		3	Missense		Pakistan	Santra et al. (2018)
c.538G>A	Asp180Asn	Elevated transaminases	1	Missense		China	Zhang et al. [23]
c.557G>A	Arg186His	Hepatomegaly, elevated transaminases, delayed growth in childhood, and elevation of serum transaminases	6	Missense	E6	Canada, USA, France, UK, Germany	Roscher et al. [28]; Burwinkel et al. [51]; Davit-Spraul et al. [29]; Hendrickx et al. [69]; Burwinkel et al. [51]
c.559 G>A	Gly187Arg	Hepatomegaly	1	Missense	E6	France	Davit-Spraul et al. [29]
c.556C>T	Arg186Cys	Hepatomegaly, delayed growth in childhood, elevated transaminases	1	Missense	E6	UK	Hendrickx et al. [69]
	Gly193Val	Hepatomegaly and growth retardation	1	Missense	E6	Japan	Hirono et al. (1995)
c.565del6	Asp189–Thr190	Hepatomegaly, delayed growth in childhood, elevated transaminases	1	Deletion	E6	UK	Hendrickx et al. [69]
c.567del22	fs189	Elevated transaminases	1	Frameshift	E6	UK	Burwinkel et al. (1998)
c.674del13	fs226	Hepatomegaly, delayed growth in childhood, elevated transaminases	1	Frameshift	E7	UK	Hendrickx et al. [69]
c.721A>G	Pro241Val	Eevere ketotic hypoglycemia	1	Missense		USA	Hodax et al. (2017)
c.749C>T	Ser250Leu	Mildly delayed psychomotor development, hypotonia, Hepatomegaly	2	Missense		Russia	Kamenets et al. [54]; Szymanska et al. (2016)
c.755C>T			1	Missense		Russia	Kamenets et al. [54]
c.772G>A			1	Missense		Russia	Kamenets et al. [54]
c.879_881del	Cys294del	Elevated transaminases	1	Deletion	E9	China	Liang et al. [26]
c.883C>G	Arg295Gly	Hepatomegaly, elevated transaminases	2	Missense	E9	China	Dong et al. [70]; Zhang et al. [23]
c.883C>T	Arg295Cys	Elevated transaminases	1	Missense	E9	China	Liang et al. [26]
c.884G>A	Arg295His	Short stature, hepatomegaly, episodes of hypoglycemia, fasting ketosis, elevated cholesterol, low-density lipoproteins, elevated triglycerides, elevated transaminases	11	Missense	E9	Russian; Hong Kong, China, South Korea	Yablonskaya et al. [20]; Ma et al. [22]; Zhang et al. [23]; Pushkov et al. [24]; Choi et al. [25]; Liang et al. [26]
c.883C>T	Arg295Ter		1	Nonsense	E9	Japan	Ban et al. [63]
c.884G>A	Arg295His	Hepatomegaly, delayed growth in childhood, elevated transaminases	4	Missense	E9	UK, China	Hendrickx et al. [69]; Lau et al. [61]
c.889G>A	Arg295His	Hepatomegaly, elevated transaminases	1	Missense	E9	China	Zhang et al. [23]
c.893G>C	Arg298Pro	Hypotonia, short stature, failure to thrive, hypoglycemic seizures, easy fatigability, elevated transaminases, and mild lactic acidosis	2	Missense	E9	USA	Qin et al. (2016); Johnson et al. (2012)
c.896 A>G	Asp299Gly	Hepatomegaly	3	Missense	E9	France, Germany	Davit-Spraul et al. [29]; Beauchamp et al. [44]; Burwinkel et al. [51]
c.897 T>G			1	Missense	E9	Russia	Pushkov et al. [24]
c.899 G>T	Gly300Val	Hepatomegaly	1	Missense	E9	France	Davit-Spraul et al. [29]
c.899 G>A	Gly300Ala	Hepatomegaly	1	Missense	E9	France	Davit-Spraul et al. [29]
c.928C>T			1	Missense	E10	India	Korula et al. [4]
c.977 G>A	Cys326Tyr	Hepatomegaly	1	Missense	E10	France	Davit-Spraul et al. [29]
c.1009delA	Ile337Ter	Hepatomegaly	1	Nonesense		UK	Beauchamp et al. [44]
	Arg352Ter		1	Nonesense		Germany	Burwinkel et al. (1998)
c.1174C>T	Arg392Ter	Hypoglycemia, elevated transaminases, hepatomegaly, glycogen storage in liver	1	Nonesense		Spain	Vega et al. [37]
c.1183-1186dup	Gly396AspfsX28	Hepatomegaly	1	Frameshift	E12	France	Davit-Spraul et al. [29]
	Pro399Ser		1	Missense	E12	Germany	Burwinkel et al. (1998)
c.1210 C>T	Gln404Ter	Hepatomegaly	1	Nonesense	E12	France	Davit-Spraul et al. [29]
c.1210delC	Gln404AsnfsX23	Hepatomegaly, elevated transaminases	1	Frameshift	E12	Canada	Roscher et al. [28]
	Glu415Asp		1	Missense	E12	South Korea	Sohn et al. (2019)
c.1245G>T	Skipping of exon 12	Hepatomegaly and elevated serum ALT	1	Splice site	E12	South Korea	Kim et al. (2020)
c.1262G>A			1	Missense	E13	Russia	Kamenets et al. [54]
c.1265dup		Ketotic hypoglycemia	1	Duplication	E13	Netherlands	Hoogeveen et al. [27]
c.1404dupT	His469SerfsTer12	Hepatomegaly, elevated transaminases, ferropenia, vitamin D deficiency; hemoglobinopathy	1	Framshift	E14	Spain	Vega et al. [37]
c.1489C>T	Arg497Ter	Hepatomegaly, hypoglycemia, elevated transaminases, vitamin D deficiency	2	nonsense	E14	Japan, Spain	Vega et al. [37]; Ban et al. [63]
c.1493C>T	Pro498Lys	Hepatomegaly	1	Missense	E14	UK	Beauchamp et al. [44]
c.1498C>T		Hepatomegaly, elevated transaminases	1	Missense	E14	China	Zhang et al. [23]
c.1561A>G	Thr521Ala	neonatal hepatosplenomegaly, onset neurological manifestations	2	Missense	E15	Canada	Smith et al. (2020)
c.1576G>A	Asp526Asn	Afebrile, mostly morning episodes of vomiting, nausea, shakiness, uneasiness, sweating, drowsiness, hypotonia, relief after food	1	Missense	E15	Denmark	Benner et a. [34]
c.1697 A>T	Ile566Asp	Hepatomegaly, growth retardation	1	Missense		Japan	Hidaka et al. (2005)
	567del22		1	Deletion		Germany	Burwinkel et al. (1998)
c.1814C>T			1	Missense		Russia	Kamenets et al. [54]
c.1925C>G	Ser642Ter	Hepatomegaly, Fatigue	1	Nonsense	E18	China	Zhang et al. [23]
c.1956insT	Asp653Ter	Hepatomegaly	1	Nonsense	E18	France	Davit-Spraul et al. [29]
c.1963G>A	Glu655Lys		1	Missense	E18	Spain	Rodríguez-Jiménez et al. [72]
c.2077A>G	Ile693Val	Hepatomegaly	2	Missense	E19	France, Russia	Savostyanov et al. [59]; Davit-Spraul et al. [29]
c.2268dup	Asp757Ter	Hepatomegaly and elevated transaminases	3	Nonesense		South Korea	Kim et al. (2020); Sohn et al., 2019; Kim et al. [9]
c.2382_2383delTC	Asn794AsnfsX15	hepatomegaly	2	Frameshift	E22	Canada	Roscher et al. [28]
c.2387_2388del	Ser796TrpfsTer13	Elevated transaminases	2	Framshift	E22	China, Spain	Liang et al. [26]; Vega et al. [37]
c.2452C>T	Gln818Ter		1	Nonsense	E22	Brazil	Sperb-Ludwig et al. [42]
c.2458 T>G	Trp820Gly	Hepatomegaly	1	Missense	E22	France	Davit-Spraul et al. [29]
c.2470C>T	Arg824Cys	Hepatomegaly, elevated transaminases	1	Missense	E22	Canada	Roscher et al [28]
c.2561insG	Leu855ProfsX87	Hepatomegaly	1	Frameshift	E23	France	Davit-Spraul et al. [29]
c.2578C>T	Arg860Trp	Frequent early morning nightterror-like agitated episodes. Agitated screaming / premature waking, relieved by food. Occasionally with vomiting	2	Missense	E23	Denmark	Benner et al. [34]; Kamenets et al. [54]
c.2606C>G	Pro869Arg	Lethargy, vomiting, sweating, shivering	4	Missense	E24	Denmark, UK	Benner et al. [34]; Beauchamp et al. [44]
c.2609delC	Pro870GlnfsTer44	Hypoglycemia, hepatomegaly, and elevated lactate	1	Frameshift	E24	USA	Wang et al. (2012)
c.2635G>T			1	Missense	E24	Russia	Kamenets et al. [54]
c.2675 A>G		Develop hypoglycemia and seizures	1	Missense	E24	Japan	Hirono et al. [31]
c.2726_2727 delTT		Hepatomegaly, Growth retardation	1	Deletion	E25	China	Zhang et al. [23]
c.2746C>T	Arg916Trp	Hepatomegaly and elevated serum ALT	7	Missense	E25	South Korea, China, France, Argentina	Kim et al. (2020); Zhang et al. [23]; Sohn et al. (2019); Davit-Spraul et al. [29]; Beauchamp et al. [44]
c.2753delG	Gly918AspfsTer2	Mild hepatomegaly, elevated transaminases	1	Frameshift	E25	Spain	Vega et al. [37]
c.2812delG	Glu938ArgfsTer6	Hypoglycemia, hepatomegaly, and elevated lactate	1	Frameshift	E25	USA	Wang et al., 2012
c.2862_2864delCCT	Leu955del	Mild hepatoesplenomegaly, elevated transaminases, microsteatosis	1	Deletion	E25	Spain	Vega et al. [37]
c.2870A>G	His957Arg	Acute gastroenteritis with dehydration, acute prerenal kidney injury, hypoglycemia, metabolic acidosis and failure to thrive	1	Missense	E26	India	Kumar et al. [55]
c.2907delT	Ser969ArgfsTer35	Elevated transaminases	1	Nonsense	E26	China	Liang et al. [26]
c.2972C>G	Gly991Ala	Hypoglycaemia and delayed motor development, Ketotic hypoglycemia	2	Missense	E27	China, Japan	Fu et al. [19]; Ago et al. [35]
c.3190C>T			1	Missense	E28	Russia	Kamenets et al. [54]
c.3308_3312dupATGTC	Leu1105MetfsTer11	Hepatomegaly, normal spleen, elevated transaminases, without hypoglycemia, normal lactate dehydrogenase and creatine kinase	2	Frameshift	E31	Vietnam	Nguyen et al. [21]
c.3210_3212del	Arg1070del	Hepatomegaly and abdominal distension	2	Deletion	E31	Finland, UK	Kim et al. (2015); Beauchamp et al. [44]
c.3208_3210delGAG	Arg1072del	Short stature, fasting hypoglycemia with ketosis, hepatomegaly, elevated transaminases	2	Deletion	E31	USA, south Korea	Morales et al. [71]; Choi et al. [25]
c.3243delC	Val1082TrpfsTer32	Elevated transaminases	1	Nonsense	E31	China	Liang et al. [26]
c.3331C>T			1	Missense		Russia	Kamenets et al. [54]
c.3339G>A	Met1113Ile	Hepatomegaly Slightly hypotonic	1	Missense	E32	Finland	Beauchamp et al. [44]
c.3349 G>A	Glu1117Lys	Hepatomegaly	2	Missense	E32	France, spain	Vega et al. [37]; Davit-Spraul et al. [29]
c.3355delAA	fs1119	Hepatomegaly, delayed growth in childhood, elevated transaminases	1	Frameshift	E32	Belgium	Hendrickx et al. [69]
c.3373G>A	Glu1125Lys	Hepatomegaly, delayed growth in childhood, elevated transaminases	3	Missense	E32	China, UK	Li et al. (2021); Hendrickx et al. [69]
c.3377C>A	Ser1126Ter	Hepatomegaly, elevated transaminases, Frequent hunger Fatigue	2	Nonsense	E32	China, Canada	Zhang et al. [23]; Roscher et al. [28]
c.3400delC			2	Deletion	E32	Czech	Rudolfova et al. [66]
c.3505C>T	Gln1169Ter	Hepatomegaly, delayed growth in childhood, elevated transaminases	2	Nonsense	E32	Japan, UK	Ban et al. [63]; Hendrickx et al. [69]
c.3507_3520del	Gln1169HisfsTer29	Hepatomegaly, elevated transaminases	1	Frameshift	E32	China	Dong et al. [70]
c.3656_3673del		Short stature, weakness, and hepatomegaly	1	Deletion	E33	Taiwan	Lin et al. [68]
c.3563_3575dupTGGAGAAAGACCA	Gln1192HisfsX15	Hepatomegaly, elevated transaminases	1	Frameshift	E33	Canada	Roscher et al. [28]
c.3597_3598del	Phe1200LeufsTer2	Elevated transaminases	1	Nonsense	E33	China	Liang et al. [26]
c.3614C>T	Pro1205Leu	Ketotic hypoglycemia, Hepatomegaly, elevated transaminases	11	Missense	E33	Netherlands, Canada, Japan, USA, China, France	Hoogeveen et al. [27]; Roscher et al. [28]; Davit-Spraul et al. [29]; Achouitar et al. [67] Wang et al. (2012); Cho et al. [30]; Hirono et al. [31]; Van den berg et al. [32]
	Gly1207Trp	Hepatomegaly, elevated transaminases, muscle hypotonia, short stature, developmental delays, and seizures, neurological symptoms	1	Missense	E33	Germany	Burwinkel et al. (1998)
c.3628G>A	Gly1210Glu	Hepatomegaly and elevated serum ALT	2	missense	E33	Czech	Rudolfova et al. [66]
c.3628G>A	Gly1210Arg	Hepatomegaly and elevated serum ALT	3	missense	E33	South Korea	Kim et al. (2020); Sohn et al. (2019); Kim et al. [9]
g.18947453-19010180del		Hepatomegaly, elevated transaminases, hypertriglyceridemia, hypoglycemia	1	Large deletion	E1-12	Spain	Herranz-Cecilia et al. [65]
c.285+2_285+5delTAGG		Hepatomegaly, elevated transaminases, hypertriglyceridemia	1	Large deletion	E3	China	Zhang et al. [23]
			1	Large deletion	E3-4	France	Davit-Spraul et al. [29]
		Hepatomegaly,	2	Large deletion	E8	South Korea	Choi et al. [25]; Park et al. [62]
		Hepatomegaly,	2	Large deletion	E18-33	South Korea	Choi et al. [25]
			1	Large deletion	E27-30	USA	Wang et al. (2012)
		Hepatomegaly, hypoglycemia	3	Large deletion	E27-33	France, South Korea	Choi et al. [25]; Davit-Spraul et al. [29]
Exon31-33del	p.[?]	Elevated transaminases	1	Large deletion	E31-33	China	Liang et al. [26]
g.18.878.700_g.18.881.544del		Hepatomegaly	1	Large deletion	I2-4	France	Davit-Spraul et al. [29]
		Hepatomegaly, elevated transaminases	1	Large deletion	I19-I26	Japan	Fukao et al. [64]
g.18.920.212_ g.18.899.044 del		Hepatomegaly	1	Large deletion	I26-33	France	Davit-Spraul et al. [29]
DelXp22.13		Ketotic hypoglycemia	1	Deletion		Netherlands	Hoogeveen et al. [27]
c.79-1G>T		Hepatomegaly, elevated transaminases	1	Splice sit		Japan	Ban et al. [63]
c.237+1G>T		Hepatomegaly	1	Splice site		China	Zhang et al. [23]
c.537+5G>A		Hepatomegaly, hyperlipidemia	1	Splice site		South Korea, France	Choi et al. [25]; Davit-Spraul et al. [29]
c.717+781		Hepatomegaly, elevated transaminases	1	Deletion	I7	South Korea	Park et al. [62]
c.718-2A>G		Hepatomegaly and elevated serum ALT	1	Splice site	I7	South Korea	Kim et al. (2020)
c.718-3C>T		Hepatomegaly	1	Splice site	I7	France	Davit-Spraul et al. [29]
c.864+225		Hepatomegaly, elevated transaminases	1	Deletion	I8	South Korea	Park et al. [62]
	Arg1038Lys	Hepatomegaly	1	Splice site	I9	China	Lau et al. [61]
c.918+1G>A		hepatomegaly and elevated ALT	3	Splice site		South Korea	Kim et al. (2020); Sohn et al. (2019); Kim et al. [9]
c.919+2A>G			1	Splice site		Russia	Savostyanov et al. [59]
c.1324+1G>T			1	Splice site	I13	Russia	Kamenets et al. [54]
c.1325-2A>G		Hepatomegaly, elevated transaminases, recurrent ketotic hypoglycemia, and short stature	1	Splice site	I13	Australia	Karande et al. [60]
c.2226+2 T>C	–	Hepatomegaly, fatty liver disease, and liver cirrhosis	1	Splice site		Iran	Beyzaei et al. [33]
c.2316-2A>C	Asn422fs	Ketotic hypoglycemia	1	Frameshift		Netherlands	Hoogeveen et al. [27]

### Genotype–phenotype correlation

The three primary clinical presentations of this condition include hepatomegaly (68%), elevated transaminases (47%), ketotic hypoglycemia (11%), and short stature (10%) [33]. Other manifestations, although rare, have also been reported, such as afebrile fasting episodes of vomiting, shakiness, uneasiness, and drowsiness, which relieve after food ingestion. Additionally, lethargy, mild motor delay, muscle weakness and fatigue, sweating, drowsiness, shakiness, vomiting, easy fatigability, mild lactic acidosis, ferropenia, vitamin D deficiency, and hemoglobinopathy have been documented [34–37].

The Arg295His variant is associated with a diverse range of clinical manifestations, including short stature, hepatomegaly, episodes of hypoglycemia, fasting ketosis, elevated cholesterol levels, increased levels of low-density lipoproteins, elevated triglycerides, and elevated hepatic transaminases. These findings suggest that the Arg295His variant is pathogenic, exerting a wide range of effects. This variant has been reported in patients from various countries including Russia (Siberian population), Hong Kong, China, and South Korea, all of which belong to the same ethnic group. Another prevalent variant, Pro1205Leu, has been associated with clinical presentations including ketotic hypoglycemia, hepatomegaly, and elevated transaminases [8, 34]. This variant has been reported in patients from diverse ethnic backgrounds, from Netherlands, Canada, Japan, the USA, China, and France.

## Phosphorylase b kinase regulatory subunit beta (*PHKB*) gene.

### Gene

The *PHKB* gene, which encodes the subunit β, is located on 16q12.1 and is responsible for autosomal recessive PhK deficiency affecting both the liver and muscle. The longer transcript variant of *PHKB* consists of 33 exons, spanning 239 kb of genomic DNA. This gene specifically encodes the beta subunit of PhK, found in both liver and muscle tissues. The degree of phosphorylation of the beta subunit plays a crucial role in determining the activity of the PhK enzyme [7].

### Variants

**Fig. 3:**
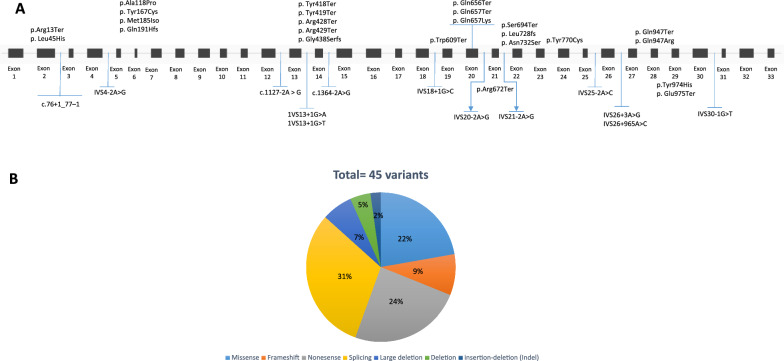
Schematic illustration (not to scale) of the *PHKB* gene and location of sequence variants associated with Glycogen storage disease type IXb deficiency. **A**: Exon (boxes)–intron (lines) structure of the human *PHKB* gene. Coloring boxes are consistent with panel **B**: Pie chart summarizing types of *PHKB* variants reported to date

Forty-five disease-causing variants in the *PHKB* gene have recently been reported in 52 patients. These variants contain different types, such as splice site (n = 14), nonsense (n = 11), missense (n = 10), frameshift (n = 4), large deletion (n = 3), deletion (n = 2), and insertion-deletion (Indel) (n = 1) (Fig. 3A, B). It appears that out of the variants identified, thirty were located in the exonic region, indicating potential functional implications for the protein-coding regions. On the other hand, fifteen variants were found in the intronic region, suggesting the importance of analyzing this region as well. The presence of variants in the intronic region may have implications for regulatory elements, such as splicing or transcriptional control. Therefore, investigating these intronic variants could provide valuable insights for the detection and understanding of patients’ conditions. It’s noteworthy that each variant identified in this study was unique, except for a specific nonsense variant called Gln657Ter, which was found in patients from two different countries, namely Macedonia and the UK [38, 39]. The majority of the reported variants have been observed in patients primarily from Europe and North America, indicating a higher prevalence in these regions. However, there have also been individual reports from Brazil, Iran, and Saudi Arabia, suggesting that the variants have been identified in diverse populations worldwide [40–43]. Understanding the distribution of these variants across different geographical locations can provide valuable insights into the global prevalence and impact of the condition associated with the *PHKB* gene (Table 3).

**Table 3:** Summary of reported variants and clinical manifestations in *PHKB* gene of patients with GSD IXb

Variant	Variant type	Clinical presentation	No. patients	Type of variant	Exon/Intron	Country	Study
	Arg13Ter		1	Nonsense	E2	Germany	Gieldon et al. (2018)
c.134 T>A	Leu45His	Hepatomegaly, short stature, and muscular hypotonia	1	Missense	E2	Iran	Beyzaei et al. [40, 45]
	Ala118Pro	Hepatomegaly	1	Missense	E6	UK	Burwinkel et al. [39]
	Tyr167Cys	Mild hepatomegaly, elevated transaminases and asymptomatic hypoglycemia	1	Missense	E6	Spain	Vega et al. [37]
c.555G>T	Met185Iso	Normal	1	Missense	E6	UK	Beauchamp et al. [44]
c.572_576delAGATT	Gln191Hfs	Hepatomegaly	1	Frameshift	E6	Brazil	Sperb-Ludwig et al. [42]
	Arg364Ter	Hepatomegaly	1	Nonsense		France	Davit-Spraul et al. [29]
	Tyr418Ter	Hepatomegaly, elevated transaminases	1	Nonsense	E14	UK	Burwinkel et al. [39]
c.1257 T>A	Tyr419Ter	Slightly hypotonic, speech poor	2	Missense	E14	Ireland	Beauchamp et al. [44]
	Arg428Ter	Doll-like face, thin extremities, hepatomegaly, and reduced muscle power and bulk	1	Nonsense	E14	UK	Burwinkel et al. [39]
	Arg429Ter	Hepatomegaly	1	Nonsense	E14	France	Davit-Spraul et al. [29]
c.1312-1322del	Gly438Serfs	Short stature, mild hepatomegaly, elevated transaminases, hypercholesterolemia and hyperuricemia	1	Frameshift	E14	Turkey	Kilic et al. [57]
c.1827G>A	Trp609Ter	Hypoglycemia, elevated transaminases, LDH, and triglycerides	1	Nonsense	E19	Natherland	van den Berg et al. [48]
	Gln656Ter	Hepatomegaly, elevated transaminases	2	Split site	E20	UK	Burwinkel et al. [39]
	Gln657Ter	Hepatomegaly, hypoglycemic episodes or muscle weakness	2	Nonsense	E20	Macedonia, UK	Zdraveska et al. [38]; Burwinkel et al. [39]
	Gln657Lys		1	Missense	E20	UK	Burwinkel et al. [46]
	Arg672Ter	Hepatomegaly	1	Nonsense	E21	-	gnomAD
c.2081C>G	Ser694Ter	Hepatomegaly	1	Nonsense	E22	Brazil	Sperb-Ludwig et al. [42]
c.2181delT	Leu728fs	Hepatomegaly	1	Frameshift	E22	Brazil	Sperb-Ludwig et al. [42]
	Asn732Ser		1	Missense	E22	Poland	Fichna et al. [77]
c.2309A>G	Tyr770Cys		2	Missense	E24	Natherland	Burwinkel et al. [46]; van den Berg et al. [58]
c.2839C>T	Gln947Ter	Hepatomegaly, elevated transaminases	2	Nonsense	E28	Canada	Roscher et al. [28]
c.2840A>G	Gln947Arg	Elevated transaminases	1	Missense	E28	Iran	Beyzaei et al. [40, 45]
	Tyr974His	Hepatomegaly	1	Missense	E29	UK	Burwinkel et al. [46]
	Glu975Ter	Hepatomegaly	1	Nonsense	E29	UK	Burwinkel et al. [39]
c.2176–255–2257+339del		Hepatomegaly	1	Deletion	E2	Turkey	Maurer et al. [43]
c.573_577delGATTA		Hepatomegaly, elevated transaminases	1	Deletion	E7	France	Davit-Spraul et al. [29]
–		Hepatomegaly, elevated transaminases	1	Large deletion	E2-E10	India	Kumar et al. [55]
		Hepatomegaly, elevated transaminases	1	Large deletion	E2-E11	India	Kumar et al. [55]
			1	Large deletion	E5-6	Saudi Arabia	Alfadhel et al. [41]
c.76+1_77–1		Hepatomegaly, elevated transaminases	2	Indel	I2	India	Kumar et al. [55]
	IVS4-2A>G	Hepatomegaly, elevated transaminases	1	Split-site	I4	UK	Burwinkel et al. [39]
c.1106-2A>G		Hepatomegaly, short stature	1	Split-site	I12	Canada	Roscher et al. [28]
c.1127-2A>G	p.?	elevated transaminases	1	Split-site	I12	Iran	Beyzaei et al. [40, 45]
	1VS13+1G>A		1	Split-site	I13	Ireland	Beauchamp et al. [44]
	1VS13+1G>T		1	Split-site	I13	Ireland	Beauchamp et al. [44]
c.1364-2A>G	–	Hepatomegaly, elevated transaminases	1	Split-site	I14	India	Kumar et al. [55]
c.1969C>T	IVS18+1G>C	Hepatomegaly, hypoglycemic episodes or muscle weakness	1	Split-site	I18	Macedonia	Zdraveska et al. [38]
c.1972‐2A>G	IVS20-2A>G	Hepatomegaly	1	Split-site	I20	Brazil	Sperb-Ludwig et al. [42]
c.2427+3A>G		Hepatomegaly, elevated transaminases	1	Frameshift		France	Davit-Spraul et al. [29]
	IVS21-2A>G	Hepatomegaly	1	Split-site	I21	Brazil	Sperb-Ludwig et al. [42]
	IVS25-2A>C		1	Split-site	I25	Ireland	Beauchamp et al. [44]
	IVS26+3A>G	Hepatomegaly, elevated transaminases	1	Split-site	I26	France	Davit-Spraul et al. [29]
	IVS26+965A>C		1	Split-site	I26	Ireland	Beauchamp et al. [44]
	IVS30-1G>T	Hepatomegaly	1	Split-site	I30	UK	Burwinkel et al. [39]

### Genotype–phenotype correlation

The reported pathogenic variants in patients are associated with a range of clinical manifestations. The most commonly observed symptoms include hepatomegaly (62%), elevated transaminases (36.5%), and muscle weakness (11.5%). In addition, there have been rare occurrences of other manifestations such as short stature, doll face appearance, slightly hypotonic muscles, speech difficulties, hypoglycemic episodes, thin extremities, and reduced muscle power and bulk [38–40, 44]. One patient with cirrhosis underwent a liver transplant due to a novel pathogenic split-site mutation that has not been reported so far. She presents with short stature, hepatomegaly, and liver cirrhosis [45]. She received liver transplantation at age 5. Understanding the diverse clinical presentations associated with these variants can help in recognizing and diagnosing the condition accurately. It appears that hepatomegaly is a prominent clinical manifestation observed in several GSD types, namely GSD I, III, VI, and IXb. Performing mutational analysis can prove to be valuable in assisting with the differential diagnosis of patients presenting with hepatomegaly. Identifying specific genetic variants associated with hepatic GSD types can help distinguish them from other potential causes of hepatomegaly and aid in providing appropriate management and treatment strategies.

However, it is important to note that due to the limited number of reported patients, it is currently not possible to establish a definitive genotype–phenotype correlation. The findings provide valuable initial insights, but further investigation and research are needed to gather more substantial evidence and draw more conclusive conclusions in this regard.

## Phosphorylase b Kinase gamma catalytic chain, liver/testis isoform (*PHKG2*) gene.

### Gene

The *PHKG2* gene is located on the 16p11.2 region and is responsible for autosomal recessive liver PhK deficiency. It consists of ten exons and spans 9 kb of genomic DNA. The longer transcript isoform of *PHKG2* encodes the catalytic gamma subunit of liver PhK, which is a protein made up of 406 amino acids [7].

### Pathogenic variants

**Fig. 4:**
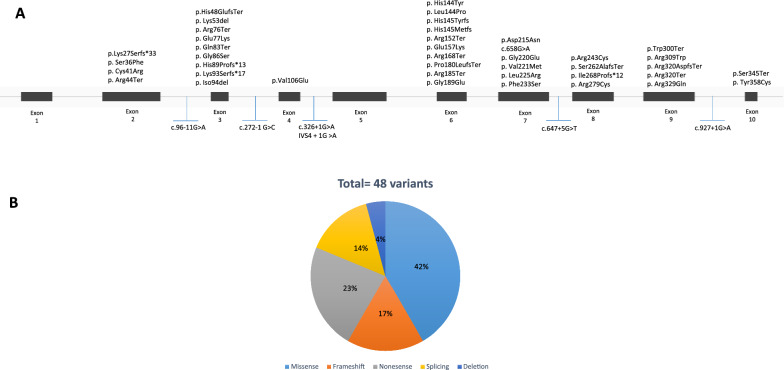
Schematic illustration (not to scale) of the *PHKG2* gene and location of sequence variants associated with Glycogen storage disease type IXc deficiency. **A**: Exon (boxes)–intron (lines) structure of the human *PHKG2* gene. Coloring boxes are consistent with panel **B**: Pie chart summarizing types of *PHKG2* variants reported to date

*PHKG2* has been associated with more than 45 pathogenic variants, including 20 missense, 11 nonsense, 8 frameshifts, 7 splice sites, and 2 deletion variants in 80 patients (Fig. 4A, B). The majority of the variants occur in the exonic region, with only seven intronic variants reported. Exons 1 and 5, however, have not shown any reported mutations so far. The all reported variants have been observed in patients from various ethnic backgrounds, spanning Europe, North America, South America, Asia, and the Middle East. The most prevalent variant observed is Glu157Lys, which has been reported in six patients from three different countries: Canada, Germany, and China [28, 46, 47]. It is important to note that one patient from Africa (Cameroon) also indicates the presence of these variants, suggesting their identification in diverse populations worldwide [29] (Table 4).

**Table 4:** Summary of reported variants and clinical manifestations in *PHKG2* gene of patients with GSD IXc

Variant	Variant type	Clinical presentation	No. patients	Type of variant	Exon/Intron	Country	Study
c.79_88delinsTCTGGTCG	Lys27SerfsTer33	Severe hepatomegaly, hypoglycemia, elevated transaminases, hypertriglyceridemia, growth retardation	1	Split-site	E2	China	Zhou et al. [78]
c.107C>T	Ser36Phe	Failure to thrive, hepatomegaly, hypoglycemic seizure	2	Missense	E2	Pakistan	Waheed et al. [49]
c.121 T>C	Cys41Arg	Hepatomegaly, elevated transaminases	1	Missense	E2	Canada	Roscher et al. [28]
c.130C>T	Arg44Ter	Hepatomegaly, short stature, elevated transaminases	3	Nonsense	E2	Iran, Saudi Arabian	Beyzaei et al. [40, 45]; Alfadhel et al.[41]; Burwinkel et al. [51]
c.144delC	His48GlufsTer	Delayed puberty, muscle weakness, fatigue, tubulopathy	1	Frameshift	E3	Pakistani	Beauchamp et al. [44]
c.158_160delAGA	Lys53del	Hypoglycemia, Hepatomegaly, elevated transaminases	1	Deletion	E3	USA	Bali et al. (2014)
c.226C>T	Arg76Ter	Failure to thrive, hepatomegaly	1	Nonsense	E3	Pakistan	Waheed et al. [49]
c.229G>A	Glu77Lys		1	Missense	E3	India	Kumar et al. [55]
c.247C>T	Gln83Ter	Hepatomegaly, hypoglycemic seizure, elevated transaminases	2	Nonsense	E3	USA, Pakistan	Bali et al. (2014); Waheed et al. [49]
c.256G>A	Gly86Ser	Hepatomegaly, elevated transaminases, failure to thrive	1	Missense	E3	Canada	Roscher et al. [28]
c.265_266insC	His89ProfsTer13	Hepatomegaly, muscular hypotonia, growth retardation, hypoglycemia	2	Frameshift	E3	Norway, Germany	Michele et al. (1998); Burwinkel et al., [46]
c.277delC	Lys93SerfsTer17	Hepatocellular adenoma, liver cirrhosis	2	Frameshift	E3	Japan	Burwinkel et al. (1998); Shiomi et al., (1989)
c.280_282delATC	Iso94del	Hypoglycemia, hepatomegaly, short stature, cirrhosis, and recurrent multiple hepatocellular adenomas	1	Deletion	E3	Japan	Kido et al. [47]
c.317 T>A	Val106Glu	Hepatomegaly, growth retardation, severe liver fibrosis, elevated ALT and triglycerides, proliferation of bile ducts	4	Missense	E4	India, Pakistan	Kumar et al. [55]; Maichele et al. (1996)
c.430 T>C	His144Tyr	Poor growth, muscle wasting, elevated transaminases, hepatomegaly	1	Missense	E6	England	Burwinkel et al. [50]
c.431 T>C	Leu144Pro	Muscle weakness and fatigue	1	Missense	E6	Pakistan	Beauchamp et al. [44]
c.432delC	His145Tyrfs	Poor growth, muscle wasting, hepatomegaly, elevated transaminases, triglycerides	1	Missense	E6	England	Burwinkel et al. [50]
c.432delC	His145Metfs	Short stature, abdominal distension, severe hepatomegaly and splenomegaly, hypoglycemia and elevated transaminases	3	Frameshift	E6	Turkey	Kilic et al. [57]
c.454C>T	Arg152Ter	Hepato-splenomegaly	2	Nonsense	E6	Brazil, Pakistan	Waheed et al. [49]; Sperb-Ludwig et al. [42]
c.469G>A	Glu157Lys	Hepatomegaly, elevated transaminases	6	Missense	E6	Canada, Germany, China	Roscher et al. [28]; Burwinkel et al. [46]; Kido et al. [47]
c.502C>T	Arg168Ter		1	Nonsense	E6	Brazil	Sperb-Ludwig et al. [42]; Davit Spraul et al. [29]
c.538Del	Pro180LeufsTer		1	Nonsense	E6	India	Kumar et al. [55]
c.553C>T	Arg185Ter	Hepatomegaly, elevated transaminases	2	Nonsense	E6	Pakistan, China	Waheed et al. [49]; Li et al. [56]
c.566G>A	Gly189Glu	Hepatomegaly, growth retardation, mild muscule hypotonia, elevated transaminases, elevated triglycerides	1	Missense	E6	France	Michele et al. (1996)
c.643G>A	Asp215Asn	Hepatomegaly, elevated transaminases	4	Missense	E7	India, Canada, Norweg	Kumar et al. [55]; Roscher et al. [28]; Burwinkel et al. [46]
c.658G>A			1	Missense	E7	Russia	Kamenets et al. [54]
c.659 G>A	Gly220Glu	Hypoglycemia, Hepatomegaly, elevated transaminases	3	Missense	E7	Saudi Arabia	Albash et al. [53]
c.661G>A	Val221Met	Hepatomegaly and elevated transaminases	2	Missense	E7	South Korea	Kim et al. (2020)
c.667 T>G	Leu225Arg	Poor growth, muscle wasting, elevated transaminases, hepatomegaly	1	Missense	E7	England	Burwinkel et al. [52]
c.698 T>C	Phe233Ser	Hypoglycaemia, growth retardation, elevated transaminases, Hepatomegaly, jaundice,	2	Missense	E7	China, India	Shao et al. (2022); Korula et al. [4]
c.727 C>T	Arg243Cys	Seizure disorder, short stature, muscle weakness, hepatomegaly	1	Missense	E8	Mexic	Kanungo et al. (2013)
c.783DelC	Ser262AlafsTer	Hepatomegaly and elevated ALT	3	Frameshift	E8	South Korea	Kim et al. (2020); Sohn et al. (2019)
c.802_805delATCT	Ile268ProfsTer12	Hypoglycemia, cirrhosis, muscular defect with distal amyotrophia, received a liver transplantation at age 20 years	1	Frameshift	E8	Comoran	Davit-Spraul et al. [29]
c.835C>T	Arg279Cys		1	Missense	E8	Brazil	Sperb-Ludwig et al. [42]
c.900G>A	Trp300Ter	Hepatomegaly, elevated transaminases	3	Nonsense	E9	USA, Germany, Norweg	Bali et al. (2014); Burwinkel et al. [46]; Michele et al. (1998)
c.925C>T	Arg309Trp	Hepatomegaly, failure to thrive, elevated transaminases	1	Missense	E9	Canada	Roscher et al. [28]
c.957insGG	Arg320AspfsTer	Jaundice, hypoglycaemia, growth retardation, elevated transaminases, hepatomegaly	1	Nonsense	E9	China	Shao et al. (2022)
c.958C>T	Arg320Ter	Hypoglycemic seizures, hepatomegaly, elevated transaminases	2	nonsense	E9	Pakistan	Waheed et al. [49]
c.986G>A	Arg329Gln	Hepatomegaly, elevated transaminases	1	Missense	E9	USA	Wang et al. (2012)
c.1034C>G	Ser345Ter	Hepatomegaly, elevated transaminases	1	Nonsense	E10	Jordon	Fahiminiya et al. (2014)
c.1073A>G	Tyr358Cys	Hepatomegaly, elevated transaminases	1	Frameshift	E10	USA	Bali et al. (2014)
c.96-11G>A		Hepatomegaly, elevated transaminases	1	Split-site	I2	USA	Bali et al. (2014)
c.272–1 G>C		hypoglycemic manifestations, progression to cirrhosis	1	Split-site	I3	France	Davit-Spraul et al. [29]
c.326+1G>A		Hepatomegaly, splenomegaly, liver fibrosis, cirrhosis	2	Split-site	I4	Turkey	van Beurden et al. [48]
IVS4+1G>A			1	Frameshift	I4	Netherlands	van Beurden et al. [48]
c.557-3C>G	–	Hypoglycemic seizure and elevated transaminases	2	Split-site	I6-7	Pakistan	Waheed et al. [49]
c.647+5G>T		Hypoglycemia, Hepatomegaly, elevated transaminases	1	Split-site	I7	USA	Bali et al. (2014)
c.927+1G>A			1	Split-site	I9	Brazil	Sperb-Ludwig et al. [42]

### Genotype–phenotype correlation

The reported pathogenic variants in patients have been associated with a diverse range of clinical manifestations. The most commonly observed symptoms include elevated transaminases (70%), hepatomegaly (68.8%), hypoglycemia (28.8%), and failure to thrive (27.5%). Additionally, there have been rare occurrences of other manifestations, such as muscular hypotonia, muscle weakness, fatigue, tubulopathy, hepato-splenomegaly, jaundice, liver fibrosis, cirrhosis, and multiple hepatocellular adenomas [44, 48, 49]. Only two patients were reported to have a hepatic adenoma and one of them was reported as receiving a liver transplant [29].

## Conclusion

This paper represents the first comprehensive literature review on variants of liver GSD IX. We aim to establish a detailed report of variants compelling evidence for a genotype–phenotype correlation across the different subtypes of liver GSD IX. Our comprehensive review reveals the unique nature of the variants found in all subtypes of GSD IX, making it challenging to establish a direct correlation between genotype–phenotype in patients. However, we have observed that individuals with GSD IXG2 generally experience a more severe clinical presentation compared to those with GSD IXA2 or B. This observation may be attributed to the fact that the γ subunit, which encompasses the catalytic site, plays a crucial role in enzyme function. Damage to this specific area, as opposed to the regulatory sites, can lead to more significant enzyme impairment, resulting in a more severe manifestation of the disease. Understanding the functional significance of different subunits and their impact on enzyme activity can provide valuable insights into the disease mechanism and help guide future therapeutic interventions. By focusing on the specific regions of the enzyme that are most critical for its function, researchers can potentially develop targeted strategies to mitigate the effects of GSD IX.

Through careful examination of these variants, we have gained valuable insights into the complex nature of GSD IX. This information will not only facilitate a better understanding of the condition but also provide a basis for further research and potential advancements in patient care. Furthermore, dedicating more research to studying the mutations and gene mapping associated with GSD IX can significantly contribute to our understanding of the intricate relationship between genotype and clinical presentation. This deeper understanding has the potential to enhance diagnosis, prognosis, and treatment strategies for affected patients. By unraveling the underlying genetic mechanisms and their impact on the clinical features of GSD IX, we can potentially pave the way for personalized medicine approaches tailored to the specific needs of individuals with this condition.

## Data Availability

All data generated or analyzed during this study are included in this published article.

## Abbreviations

GSD: Glycogen storage disease
PhK: Phosphorylase kinase
PHKA1: Phosphorylase b Kinase regulatory subunit alpha
PHKA2: Phosphorylase b Kinase regulatory subunit alpha 2
PHKB: Phosphorylase b Kinase Regulatory Subunit Beta
PHKG2: Phosphorylase b Kinase gamma catalytic chain
